# Cortical Potentiation in Chronic Neuropathic Pain and the Future Treatment

**DOI:** 10.3390/ph18030363

**Published:** 2025-03-04

**Authors:** Shun Hao, Shen Lin, Wucheng Tao, Min Zhuo

**Affiliations:** 1 Key Laboratory of Brain Aging and Neurodegenerative Diseases, Fujian Medical University, Fuzhou 350122, China; haoshun911@126.com (S.H.); taowucheng@fjmu.edu.cn (W.T.); 2 Fujian Provincial Institutes of Brain Disorders and Brain Sciences, First Affiliated Hospital, Fujian Medical University, Fuzhou 350005, China; 0621133@zju.edu.cn; 3 Department of Physiology, Faculty of Medicine, University of Toronto, Medical Science Building, 1 King’s College Circle, Toronto, ON M5S 1A8, Canada

**Keywords:** neuropathic pain, chronic pain, ACC, IC, thalamus, synaptic plasticity, LTP, LTD, pre-LTP, post-LTP, synaptic tagging, descending facilitation, AMPAR, NMDAR, KAR, AC1, PKMζ, HCN channel, ASIC1a, CGRP, oxytocin, BDNF, TBS, LFS, analgesic, treatment

## Abstract

Pain, or the ability to feel pain and express the unpleasantness caused by peripheral injuries, are functions of the central nervous system. From peripheral sensory nerve terminals to certain cortical regions of the brain, activation of related neural networks underlies the sensory process. Recently, our knowledge of pain has been increasing dramatically, due to the advancement of scientific approaches. We no longer see the brain as a random matrix for pain but, rather, we are able to identify the step-by-step selective signaling proteins, neurons, and networks that preferentially contribute to the process of chronic pain and its related negative emotions, like anxiety and fear. However, there is still lacking the selective and effective drugs and methods for the treatment of chronic pain clinically. While first-line drugs for acute pain and mental diseases are also applied for the clinical management of chronic pain, their prolonged usage always causes serious side effects. In this short review, we will update and summarize the recent progress in this field and mainly focus on the roles of neural networks and synaptic mechanisms in chronic neuropathic pain. Furthermore, potential drug targets (such as plasticity-related signaling molecules, ionic channels, cytokines, and neuropeptides) and methods for the management of chronic neuropathic pain will be discussed as well. We hope this review can provide new, valuable insight into the treatment of chronic neuropathic pain.

## 1. Introduction

**Figure 1:**
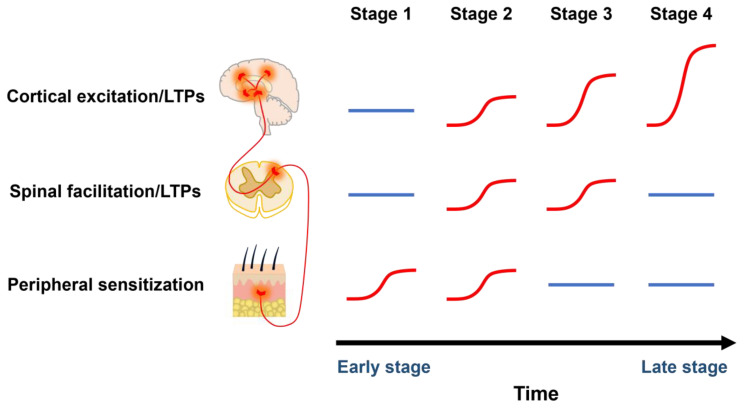
The changes of peripheral and central neural plasticity at different stages of chronic pain. Peripheral sensitization, spinal cord facilitation and sensitization, as well as cortical plasticity contribute to pain perception at different stages of chronic pain. At the early stage of chronic pain after peripheral injury, peripheral and spinal cord sensitization plays an important role in hyperalgesia and allodynia. At the late stage, cortical synapses are also likely undergoing long-term potentiation (LTP). For certain forms of chronic pain, because of cortical excitation and LTPs, patients still complain about pain even after the full recovery of the peripheral injury.

Neuropathic pain, as a kind of classic and common chronic pain, is always caused by the lesions of peripheral nerves, such as the ones in our organs, arms, legs, and fingers, or by direct injury to the central nervous system (CNS) [1]. Cumulative evidence has consistently suggested that acute pain and chronic pain are different in terms of central regulatory mechanisms. While some basic neuronal networks may share between acute and chronic pain, long-term plastic changes in the CNS may selectively contribute to chronic pain at different stages (Figure 1). At the early stages of peripheral injury, peripheral sensitization first activates the spinal cord and somatosensory cortices, causing hyperalgesia and allodynia. Following long-lasting stimuli from the peripheral injury, the pain-related cortices are always under a excitatory situation, inducing long-term potentiation (LTP) of the cortical synapses at the late stage. Spontaneous pain and allodynia are likely triggered by ongoing brain activities or sensory stimuli that are not painful at all before the injury. In this case, patients continuously suffer long-term pain despite the complete recovery of the peripheral injury. The previous failure of many CNS drugs for treating chronic pain was partially caused by the use of acute pain mechanisms for designing the targets for chronic pain. It is unavoidable that such pain medicines, like gabapentinoids and tricyclic antidepressants as the first-line drugs for treating chronic pain, will have many side effects (such as lethargy, vertigo, peripheral swelling, suicide risk, etc.) if they are used for a long period [2]. Therefore, we need to urgently explore potential pathogenic mechanisms to find and develop new targets and methods for the improvement of the diagnosis and treatment of chronic pain.

## 2. Spinal Sensitization: Re-Enforce Positive Feedback in Chronic Neuropathic Pain

In the CNS, the spinal cord acts as the primary center of afferent nociceptive processing. Long-term central sensitization in the spinal cord is also an important reason for chronic pain. After an injury, spinal nociceptive transmission is enhanced and sensitized at the synaptic and neuronal level (i.e., increases in firing action potentials) [3,4]. These excitations will lead to ascending nociceptive information into various subcortical regions and finally hyperactivate cortical regions that are responsive to nociceptive inputs and pain perception [5]. In addition, it has been found that cortical neurons can send descending projections back to the spinal cord, through direct projections or relays through the brainstem, to further enhance nociceptive inputs at the spinal level [6,7]. Thus, spinal sensitization serves as a key relay for such positive feedback and ensures that ascending nociceptive transmission is ongoing and maintains cortical excitation for a long time.

## 3. Brain Regions and Neural Networks Related to Pain Perception and Neuropathic Pain

At the supraspinal level, there are many subcortical and cortical areas involved in pain perception and chronic pain. Accumulative evidence from human and animal experiments shows that there is a pain matrix in the brain [8]. This pain matrix consists of the subcortical areas (such as the thalamus, periaqueductal grey (PAG), parabrachial nucleus (PB), amygdala) and cortical areas (such as cingulate cortex (CC), prefrontal cortex (PFC), insular cortex (IC), and primary/secondary somatosensory cortex (S1/S2)). All these brain regions connect with each other and set up a neural regulatory network of pain and pain-related emotions [5,9]. A previous EEG study identified many chronic pain-related cortical regions in a population of neurogenic pain patients. The increased theta and beta rhythms were localized to multiple pain-associated areas, primarily to IC, PFC, the anterior cingulate cortex (ACC), and inferior posterior parietal cortex (PPC), as well as to S1, S2, and the supplementary somatosensory (SSA) cortex. One year after a thalamic therapeutic surgery (central lateral thalamotomy, CLT), the theta and beta overactivation in the CC and IC of the patients was significantly reduced [10]. These findings are consistent with the results obtained from other methods like positron emission tomography (PET) and functional magnetic resonance imaging (fMRI) in patients [11,12,13].

### 3.1. Roles of Thalamus in Neuropathic Pain

In accordance with numerous related studies, our attention focuses on three brain regions, including the thalamus, ACC, and IC [5,9]. These regions are tightly associated with neuropathic pain and have great potential for the treatment of chronic pain as promising targets in the brain. The thalamus, as a key relay for sensory information to the cortex, is also a subcortical center for pain modulation. The thalamic function in acute pain is well known, but the roles of the thalamus, especially various thalamic subnucleus, in chronic pain are still under investigation. The thalamus receives the nociceptive inputs from the periphery and communicates with different cortical regions bidirectionally [9]. Many imaging studies have suggested that episodes of spontaneous pain have a significant correlation with changes in patients’ thalamic activity and regional cerebral blood (rCBF) flow [14,15]. In patients with chronic back pain (CBP), the CBP responses are correlated with bilateral activity in the posterior thalamus [16]. Another recent study reported that spinal cord injury in a FosTRAP2 reporter mouse line led to the altered activities of various supraspinal pain-related neuron populations. The results showed a large increase in neural activation in the thalamus and a significant decrease in the PAG, an important descending inhibitory center of pain transmission [17]. In addition, some functional brain imaging studies have described the decreased volume of the thalamus and altered connections between the thalamus and different cortical regions, such as the IC and amygdala, related to pain processing in patients with chronic neuropathic pain [15,18,19,20,21]. Hence, all the available evidence suggests that the thalamus contributes to chronic pain states but the function of every subnucleus in detail remains unclear and needs further investigation.

### 3.2. Roles of ACC and IC in Neuropathic Pain

At the cortical level, accumulative studies from humans and animals have consistently confirmed that two cortical regions, the ACC and IC, play important roles in nociceptive perception and chronic pain [5,9,22,23,24]. The ACC and IC have been found to respond to various peripheral nociceptive stimuli (such as noxious cold/heat and chemical stimuli). In chronic pain models, it was reported that activity-dependent immediate early genes were activated in the ACC and IC after peripheral nerve injury or inflammation. In the inflammatory pain model, adenylyl cyclase subtype1 and 8 (AC1 and 8) genetic knockout mice showed significantly reduced pain responses and expression of immediate early genes in the ACC and IC [25,26,27]. Electrophysiological and calcium imaging experiments offered direct evidence that ACC and IC neurons could be activated by noxious stimuli, and the majority of these neurons belonged to excitatory pyramidal neurons [25,28,29]. In addition, activating the ACC contributed to freezing responses in the fear memory test, supporting the involvement of the ACC in pain-related unpleasantness [30]. In human brain imaging studies, it has been discovered that psychological pain and social exclusion also activate the ACC and/or IC regions, providing additional evidence for their significance in the pain process [22,31].

Recently, more efforts have been devoted to pain-related circuit connections and their functions of the ACC and IC. A human fMRI study showed that the analgesia induced by δ-9-tetrahydrocannabinol (THC), the main psychoactive component in cannabis, was correlated with the reduction of functional connectivity between the ACC and sensorimotor cortex in patients with chronic radicular neuropathic pain. The network connectivity in some pain-related areas, such as the dorsolateral prefrontal cortex, was reduced as individual pain was alleviated [32]. Li et al. identified the medial prefrontal cortex (mPFC)^Glu^-ACC^GABA^ projections as an analgesic cortical–cortical pathway. Activating this pathway attenuated pain-like behaviors in nerve injury mice [33]. In the ACC, one side of the ACC can receive glutamatergic projections from the contralateral side. The activation of this connection crossing the corpus callosum facilitated pain responses to nociceptive stimuli in mice. On the contrary, the inhibition of this pathway produced an analgesic effect in the neuropathic mice [34]. Song et al. identified an ACC-ventral tegmental area (VTA)-ACC positive-feedback pathway which mediated the progress and maintenance of neuropathic pain and anxiodepressive-like behavior in mice [35]. In addition, similar studies about the IC-mediated pathways of pain modulation spring up as well. In patients with diabetic peripheral neuropathy, after stopping clinical pain treatment, fMRI data showed that thalamocortical functional connectivity between the left thalamus and IC/S1 increased [36]. It has also been reported that the neuropathways from the posterior insular cortex to BLA and ventromedial thalamic nucleus contributed to hyperalgesia and depression-like behavior in mice with spared nerve injury (SNI) [37]. Furthermore, much evidence emphasizes the significance of IC in trigeminal neuropathic pain [19,38,39]. It reported a descending pathway from the IC to the trigeminal caudal subnucleus (Vc) in the modulation of chronic orofacial pain [39]. These results suggest the comprehensive involvement of ACC and IC and their related neural network in chronic neuropathic pain.

At the synaptic level, anatomic and biochemical studies in animals indicate that peripheral or central nerve injuries alter synaptic structures and related signaling pathways, inducing long-term synaptic changes in the ACC and IC [5,40,41]. This reinforced central sensitization is always modulated by the expression and translocation of pre/postsynaptic neurotransmitter receptors, ion channels, synaptic proteins, and the activation of their related up/downstream signaling pathways [5,40,42]. Recently, the neural cell adhesion molecules have also been recognized as the important modulators in synaptic plasticity [41,43]. Ko et al. (2018) found that peripheral nerve injury activated the ACC and enhanced the turnover of specific synaptic proteins, neural cell adhesion molecule 1 (NCAM1), in a persistent manner [41]. The increased NCAM1 turnover in the ACC mediates spine reorganization and mechanical sensitized responses, providing a similar mechanism with the maintenance of N-methyl-D-aspartate-receptor (NMDAR)- and protein-synthesis-dependent long-term synaptic changes in the ACC. Another study revealed that laminin functioned in ACC-mediated neuropathic pain. Laminin β1 (LAMB1) in the ACC was significantly reduced after peripheral injury. LAMB1 knockdown was seen in ACC-induced allodynia and pain-related anxiodepression. This effect was caused by increasing local presynaptic release of neurotransmitters and abnormal postsynaptic spine remodeling in ACC pyramidal neurons [43]. These studies suggest that extracellular matrix aberrations likely raise a new insight into the treatment of neuropathic pain.

Meanwhile, it must be noted that not all cortical areas positively contribute to pain perception. Recent studies in the prefrontal cortex (PFC) reveal that neurons in the PFC project to the midbrain periaqueductal gray (PAG), and activate well-known endogenous analgesic systems to control pain [44]. This assumes that different cortical areas are involved in pain perception, unpleasantness, and subsequently the inhibition of such pain transmission at lower parts of the body. In summary, the dysfunctions of different neural pathways in the CNS contribute to different behavioral and emotional components of neuropathic pain, respectively. Systematic connectomics and functional studies are essential to explore the etiology of neuropathic pain mediated by various neural pathways, especially those associated with the thalamus, ACC, IC, S1/2, PFC, and amygdala.

## 4. Long-Term Potentiation (LTP): A Key Synaptic Mechanism for Central Sensitization

**Figure 2:**
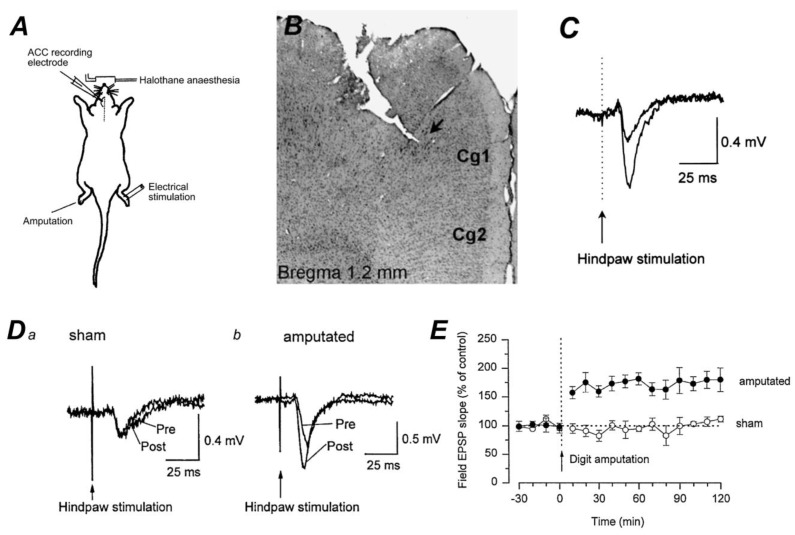
In vivo LTP in the ACC induced by peripheral single digit amputation in adult rats. (**A**) Diagram of in vivo fEPSP recording in the rat ACC; The rat was kept in a mildly anesthetized condition using halothane. The recording electrode was positioned in the opposite ACC to the peripheral stimulation electrode. Amputation was conducted on the hindpaw that did not receive stimulation by applying a higher concentration of halothane. (**B**) An example of a histological section showing the recording site (arrow) labeled with neurobiotin and the track of the recording electrode. (**C**) Sample traces of synaptic responses to electrical stimulation were applied to the hindpaw at a low intensity (5.0 V) and a higher intensity (25.0 V). An arrow indicates the time of hindpaw electrical stimulation. (**D**) The representative traces of EPSPs recorded 5 min before (pre) and 115–120 min after (post) sham treatment (Figure **Da**) or amputation (Figure **Db**) in the ACC. (Figure **Db**) The latency of evoked responses remained unchanged after amputation whereas there was a notable increase in the slope of the EPSP. (**E**) Amputation of the contralateral hindpaw caused LTP response of fEPSPs in the ACC (•). The fEPSP slopes were not significantly changed in the sham group. (○) (Adapted from Wei and Zhuo, *J Physiology*, 2001 [26]). Cg1, cingulate cortex area 1; Cg2, cingulate cortex area 2.

LTP and long-term depression (LTD) are two forms of synaptic plasticity that have been widely studied in the context of learning and memory. Chronic pain can be considered a kind of persistent painful memory. Increasing evidence suggests that LTP in the CNS is a key synaptic mechanism for central sensitization [5,45,46,47]. There are different forms of LTP in both ACC and IC reported. Figure 2 shows an example of in vivo LTP recorded in the ACC induced by digit amputation. Subsequent studies reveal that LTP can be divided into at least two major forms: post-LTP is postsynaptically induced and expressed; pre-LTP is presynaptically induced and expressed. Depending on the time course recorded, LTP can be sorted into early-phase LTP (E-LTP) and late-phase LTP (L-LTP) as well [47]. L-LTP is more sensitive to inhibition of protein synthesis [48]. LTP in the ACC and IC can be effectively induced by applying several electrical stimulation protocols, like spike-timing, theta-burst stimulation (TBS), and pairing protocols [5,48,49]. Another way to induce LTP is the chemical bath application of substances such as glycine, calcitonin gene-related peptide (CGRP), or brain-derived neurotrophic factor (BDNF) [40].

For post-LTP, the activation of postsynaptic NMDARs is essential for the induction in the ACC. Bath application of NMDAR inhibitor AP-5 effectively blocks the induction of post-LTP via different protocols in the ACC. Meanwhile, using a selective GluN2A/2B antagonist can significantly inhibit the induction of post-LTP, indicating its importance in the induction [50]. This activation results in the influx of Ca^2+^ through NMDARs and a subsequent release of Ca^2+^ from intracellular stores [5,51]. Postsynaptic injection of BAPTA, a selective chelator for calcium, completely blocks the induction of LTP in the ACC, suggesting critical roles of postsynaptic Ca^2+^ in the induction of LTP [50]. Besides triggering Ca^2+^ influx, postsynaptic NMDARs activate a series of intracellular signaling pathways, including AC1, calmodulin (CaM), protein kinase A (PKA), cyclic AMP (cAMP), and immediate early genes etc. [51]. The expression of ACC LTP requires the involvement of the 2-amino-3-(3-hydroxy-5-methyl-isoxazol-4-yl) propanoic acid receptor (AMPAR) subtype 1 (GluA1). A recent study indicated that calcium-permeable GluA1s were closely involved in the PKA-dependent form of LTP. The phosphorylation site of GluA1 mediated by PKA at serine 845 is required for the expression of LTP in the ACC [52]. In addition, protein kinase M zeta (PKMζ) is essential for the maintenance of LTP as well. The application of a PKMζ inhibitor, zeta inhibitory peptide (ZIP), has been shown to completely abolish LTP in the ACC. Microinjection of ZIP into the ACC obviously reduced mouse behavioral sensitization in the peripheral nerve injury model, indicating the importance of PKMζ for ACC-mediated LTP and associated neuropathic pain [53]. Recently, Li et al. demonstrated that acid-sensing ion channel 1a (ASIC1a) in excitatory neurons of ACC contributed to cortical TBS-induced LTP and chronic pain. ASIC1a-mediated cortical plasticity relied on protein kinase C λ (PKCλ), which enhanced membrane trafficking of GluA1s in the ACC. Specifical blockade of ASIC1a by genetic deletion or pharmacological antagonists in the ACC inhibited the induction of LTP and rescued inflammatory thermal and mechanical hypersensitization in male mice with chronic pain models [54].

Although studies about LTP in the IC are few, it is likely that analogous synaptic mechanisms are engaged in this process [23,55,56]. Using a MED64 multi-channel field potential recording system, Liu et al. found that TBS induced both E-LTP and L-LTP for over 3 h in the IC of mouse brain slices. Activation of both GluN2A and GluN2B subunits of the NMDARs, metabotropic glutamate receptors (mGluRs), or L-type voltage-gated calcium channels (L-VGCCs) is essential for the induction of protein synthesis-dependent LTP in the IC. The expression of L-LTP required the involvement of postsynaptic calcium-permeable AMPARs (CP-AMPARs) [55]. Another study showed that the induction of LTP by the pairing protocol was postsynaptic NMDAR-dependent in the IC. Application of AP-5 could completely block the LTP induction [56]. Postsynaptic application of BAPTA blocked the induction of IC LTP completely, indicating the importance of postsynaptic calcium. Furthermore, calcium-activated AC1 but not AC8 was required for this potentiation as well. Inhibition of CP-AMPARs or PKMζ reduced the expression of LTP. These results suggest that AC1 can be a key modulator for the induction and maintenance of LTP in the IC. In addition, accumulative evidence showed that BLA stimuli could induce LTP in the IC, which contributes to conditioned taste aversion (CTA) and related memory [57,58,59,60]. There is a similar regulatory mechanism of LTP in the BLA-IC pathway, like that in the ACC. Many LTP-related molecules, such as calcium/calmodulin-stimulated protein kinase II (CaMKII), NMDARs, BDNF, and extracellular regulated kinase 1/2 (ERK1/2), have been proven to be involved and probably associated with IC-mediated pain processing as well. Strikingly, a new study in 2025 has reported an artificial induction of LTP in the rat IC by repetitive optogenetic stimulation of parvalbumin-immunopositive neurons (PVNs) in a manner like TBS. This LTP can increase the amplitude of IPSCs for more than 50 min and attenuate orofacial pain behaviors [61]. It raises a viable method for the treatment of chronic pain by manipulating the plasticity of inhibitory neurons. Compared with ACC, it requires much more work to enrich our knowledge about the mechanisms of LTP induction and maintenance in the IC. At the same time, further studies about the roles of various cortical neurons with different neurotransmitters/modulators expressing and sites in synaptic plasticity should get more attention no matter whether in the ACC and IC or other pain-related cortical regions.

## 5. Presynaptic LTP (Pre-LTP) and Pain-Related Emotions

In addition to post-LTP, pre-LTP can be evoked and recorded through paired-pulse low-frequency stimulation (LFS) and is always recognized as an NMDAR-independent form of LTP [62]. In recent years, pre-LTP has been a key form of synaptic plasticity to explain the mechanisms of negative emotions, especially pain-related anxiety [62,63,64]. Presynaptic receptors and related downstream signaling pathways are tightly associated with the expression of pre-LTP. For example, presynaptic kainate receptors (KARs) are critical for pre-LTP induction in mice. Genetic deletion of the GluK1 subunits in the mice blocked the expression of pre-LTP in the ACC. The application of a selective GluK1 antagonist, UBP31060, can also block pre-LTP. Unlike the NMDAR-mediated LTP, KAR-mediated pre-LTP depends on the increased probability of presynaptic neurotransmitter release, which is related to the anxiety-like behaviors induced by chronic pain [62]. A similar regulatory mechanism of pre-LTP has also been reported in the IC [64]. Hyperpolarization-activated cyclic nucleotide-gated (HCN) channels are also demonstrated to participate in the modulation of pre-LTP via a persistent depolarization of presynaptic terminals, increasing the probability of release. Additionally, the induction of pre-LTP in the ACC also requires the activation of the cAMP signaling pathway. For instance, Ca^2+^-stimulated AC1 is essential for ACC pre-LTP [62]. In the IC, AC1 also contributes to the induction of pre-LTP. Genetically knocking out AC1 or applying AC1 inhibitor NB001 blocked the pre-LTP in the IC [65]. Koga et al. combined LFS paired with a GluK1-containing KAR agonist to induce ACC pre-LTP in the WT mice. However, this pre-LTP disappeared in Fmr1 KO mice, indicating that fragile X mental retardation protein (FMRP) signaling also plays a key role in the KAR-dependent pre-LTP in the ACC [63]. A recent study has identified SCRAPPER, an E3 ubiquitin ligase expressed in presynaptic terminals, as a novel target for regulating the pre-LTP in the ACC. In the ACC of SCR-KO mice, the frequency and amplitude of the miniature and spontaneous excitatory were selectively increased. Meanwhile, the pre-LTP was completely blocked [66]. In any case, the key diffusible retrograde messenger NO is crucial for homosynaptic pre-LTP in the IC pyramidal neurons. Application of the NO synthase (NOS) inhibitor L-NAME can abolish the maintenance of pre-LTP in the IC. Meanwhile, this induction of pre-LTP is also KAR-dependent [67].

Interestingly, pre- and post-LTP can be observed in the same neurons, presumably at the same excitatory synapses [51,62]. This functional overlap probably contributes to the interaction between behavioral and emotional expression, such as, a bad emotion enhancing the existing pain feeling, and vice versa. Another clinical implication is that this interaction provides an explanation for the validity of psychotropic drugs in the treatment of chronic pain [51]. Therefore, further understanding of different forms of plasticity like pre/post-LTP in different brain regions will help us develop new strategies to treat chronic pain and its related emotional disorders.

## 6. Long-Term Depression (LTD): A Key Mechanism to Reverse Neural Excitation

LTD has been proposed as a key synaptic mechanism to counterbalance LTP. Resetting synaptic strength allows the same synapses to be involved in subsequent or recurring learning, at least in a simple hypothetical way. LTD, as another type of inhibitory synaptic plasticity, has also been demonstrated to function in the regulation of chronic pain [5,68]. There are two major forms of LTD observed in the ACC and IC according to the dependence of NMDARs. One form of NMDAR-independent LTD requires the activation of mGluR1 and L-VGCCs but not NMDARs. The other form of NMDAR-dependent LTD requires the activation of GluN2A and GluN2B subunits and increases intracellular Ca^2+^ and CaM levels at post-synapses [40,51]. Stimulation protocols for the induction of LTD with a lower frequency (1 Hz) and longer duration (over 15 min) compared to LTP can produce LTD effectively in the ACC, IC, and hippocampus [51,69,70,71,72,73,74].

On the one hand, NMDAR-dependent LTD induced by low-frequency stimulation in the ACC requires the involvement of not only NMDARs but also AMPARs. In whole-cell patch-clamp recording, presynaptic 1 Hz stimulation paired with postsynaptic depolarization (−45 mV) induced NMDAR-dependent LTD in the ACC [75,76]. The activation of GluN2A/2B subunits and the following postsynaptic Ca^2+^ influx are essential for the NMDA-dependent LTD [76]. In the mouse bone cancer pain model, NMDAR-dependent LTD in the ACC was inhibited due to a significantly decreased expression of GluN1, GluN2A, and GluN2B subunits [77]. Similarly, in another chronic neuropathic model with constriction injury of the sciatic nerve, the spike-timing dependent LTD in the ACC was seriously impaired and even persisted after injury recovery [78]. Another study reported that AMPAR GluR2s but not the GluR3s subunit were critical in the rapid induction of ACC LTD. LTD in the ACC was abolished in GluR2 knockout mice. Furthermore, pharmacological results showed that postsynaptically inhibiting GluR2-PDZ interactions blocked the induction of LTD in a 5-min time window after the induction of LTD, indicating the rapid involvement of GluR2-PDZ interactions in NMDAR-dependent LTD induction [76]. At this time, the interaction between NMDARs and NMDAR-dependent LTD is still unclear, and the more refined regulatory mechanism requires further investigation.

On the other hand, the performance of NMDAR-independent LTD in the ACC is more related to mGluRs, L-VGCCs and AMPARs [73]. Peripheral amputation of the distal tail impaired LTD in the ACC for more than 2 weeks. During the priming phase, activating mGluR1 receptors by co-applying (RS)-3,5-dihydroxyphenylglycine (DHPG) and MPEP could rescue the LTD impairment caused by neuropathic pain. The process of this metaplasticity required the involvement of PKC. Sequentially applying chelerythrine (a PKC inhibitor) during the priming period abolished the LTD rescue mediated by DHPG and MPEP in the ACC of the mice with peripheral amputation. The application of either nimodipine (L-VGCC antagonist) or MCPG (mGluR1 antagonist) completely blocked LTD. The impairment of L-VGCCs on presynaptic terminals may inhibit the effects of LFS stimulation by reducing the activity of AC1 as well as neurotransmitter vesicle formation and release [73]. These results suggest that LTD impairment after injury in the ACC may contribute to cortical excitation, in addition to the generation of LTP. Recently, Wang et al. have identified an interaction between Caspase 3 (Casp3) and AMPAR subunits which regulates the internalization of AMPARs and LTD induction in the ACC. Disrupting this interaction inhibits LTD induction and leads to peripheral pain hypersensitivity, while overexpression of cingulate Casp3 rescues LTD induction and produces analgesia in mice with nerve injury [79].

A similar form of LTD has been reported in the IC. The induction of LTD in the IC required the activation of NMDARs, mGluRs, and L-VGCCs. The endocannabinoid signaling system and protein phosphatase 1/2A, but not PKC, PKA, PKMζ, or CaMKII, are essential for the LFS-induced LTD in the IC [74]. There is another form of NMDAR-independent LTD induced by the application of DHPG in the IC. This chemically induced LTD could overlap with electrically induced LTD, suggesting that different forms of LTD may coexist within the same synaptic population. The study identified many key molecules involved in the induction of LTD in the IC [74]. Liu and Zhuo (2014) found that tail amputation selectively occluded LFS-induced LTD but not chemically DHPG-induced LTD in the IC of adult mice [80]. The occluded LTD could be induced again via the use of a lower dose of DHPG. The process of this metaplasticity requires the involvement of PKC, not PKA or CaKMII.

All of these findings provide important regulatory mechanisms of LTD in cortical regions. In models of chronic pain, impaired LTD allows pain-related cortical networks to maintain dysregulated overactivation, causing the persistence of pain. Restoration of impaired LTD in chronic pain by manipulating LTD-related core elements, such as mGluRs, PKC, L-VGCCs and GABAergic transmission, probably brings up a new therapeutic strategy to resolve the imbalance of abnormal synaptic transmission and improve the symptoms in neuropathic pain.

## 7. Synaptic Tagging: Possible Mechanism for Pain-Related Disorders

The concept of synaptic tagging first came out in the hippocampus. It describes a phenomenon that a weak stimulation is enough to induce L-LTP at a synapse when preceded by a stronger stimulation from another pathway converging on the same population of neurons [81]. The Synaptic Tagging and Capture hypothesis is always used to elucidate the storage and allocation mechanism in learning and memory [82]. Synaptic tagging is a heterosynaptic form of LTP that belongs to L-LTP [83]. L-LTP induction composes two separate processes, including a dendritic tag that marks the stimulated synapse and a molecular cascade that produces new plasticity-related mRNAs and proteins. Tagged synapses tend to undergo L-LTP with a weak stimulus that normally does not cause L-LTP [84,85]. Additionally, evidence showed that there were temporal constraints between strong and weak stimulations in synaptic tagging. The interval has a negative correlation with the priming effect of the strong stimulation [86]. By using a MED64 multi-channel field potential recording system in the ACC, Liu et al. found that a weak TBS, which only caused E-LTP or No-LTP normally in most activated channels, induced L-LTP when a strong TBS from another stimulation site was given earlier to tag the same neurons within a certain time window. Synaptic tagging both in the hippocampus and ACC is dependent on the synthesis of new plasticity-related proteins [83]. It has been reported that peripheral injuries such as tail amputation can abolish synaptic tagging in the ACC of adult mice [83]. A reasonable explanation is that the cortical potentiation causing from peripheral injury results in a ceiling state in which synaptic transmission cannot be enhanced any more [40]. Interestingly, a recent study revealed that synaptic tagging could be biphasic. The tagging of LTD was observed in the ACC of adult mice [87]. It requires the activation of mGluR1 and L-VGCCs. Application of the mGluR1a antagonist LY367385, mGluR1b antagonist CPCCOEt or L-VGCC blocker nimodipine erased the induction of tagged LTD. This induction is time-related with the best temporal constraint between two independent stimuli less than 30 min. In the tail amputation model, tagged LTD was either reduced or completely blocked. These results confirmed the existence of a memory-related synaptic tagging-like phenomenon in the ACC, indicating the loss of synaptic tagging may account for injury-related cognitive impairments and phantom pain. It also allows other non-painful events, such as fear memory and PTSD, to form pathological associations with chronic pain. Besides, many candidate proteins have been proposed to contribute to the maintenance of tagging in the hippocampus, such as CaMKII, β-actin, CP-AMPARs, Substance P, and PKA [82,85,88,89]. They are good candidates for subsequent related studies in other brain regions. Table 1 summarizes different forms of synaptic potentiation reported in pain-related ACC and IC.

## 8. Top-Down Descending Facilitatory Modulation

Spinal sensory transmission, including nociceptive transmission, is well demonstrated to receive descending biphasic modulation from supraspinal structures. In the rostroventromedial medulla (RVM), the implementation of electrical or chemical stimulation inhibits or facilitates the responses of spinal dorsal neurons and spinal reflexes to nociceptive stimuli. In pathological pain conditions, the descending facilitatory pathway often exhibits abnormal activation or enhancement [90]. However, previously, most attention was put on descending modulation from the brainstem. There is little direct evidence supporting direct corticospinal modulation in nociceptive processing at the neuronal level. Recently, Chen et al. found that selective activation of direct projecting neurons from the ACC to the spinal cord enhanced excitatory synaptic transmission and elevated intracellular Ca^2+^ levels in the spinal dorsal horn neurons, causing the sensitization of pain responses [7]. This top-down modulation is recognized to be through direct cortical–spinal projections, without relay in the brainstem. This direct cortex–spinal modulation forms a new positive feedback circuit that enforces spinal excitatory synaptic transmission and pain perception for a long time in neuropathic pain, occluding ACC-spinal top-down facilitation. Given the quick development of brain connectomics, more studies about corticospinal projections and their functions in somatosensory processing are required in the future.

## 9. Glial Cells (Microglia and Astrocytes) in Pain-Related Plasticity and Chronic Pain

At the level of the spinal cord, both astrocytes and microglia, two major glial cell types in the CNS, are found to play important roles in chronic pain-related changes [91,92,93]. The process of neuropathic pain always follows with the activation of astrocytes and microglia in the CNS, producing local pro-inflammatory and neuropathological responses, such as chronic inflammation, neuronal hyperexcitability and neurotoxicity [91,94]. These pathological effects depend on the interactions between glial cells and pain-coding neurons and usually amplify pain perception by the release of inflammatory factors, neurotransmitters, and neurotrophic factors under chronic pain conditions [94,95,96]. However, their possible roles in chronic pain, especially in the ACC and IC remain to be determined. Unlike glial cells in the spinal dorsal horn, peripheral injury failed to cause obvious changes in the ACC and IC [91,97]. Both post-LTP and pre-LTP in the ACC, two key forms of synaptic plasticity for chronic pain and its related anxiety, are insensitive to the microglial inhibitor minocycline, indicating that microglia have little effect on LTP in the ACC [5,98]. At synaptic levels, it has been reported that microglial cells recorded at the spinal and supraspinal levels are independent of synaptic plasticity, suggesting that microglial cells are unable to code incoming nociceptive information triggered by peripheral injuries as neurons did [99,100]. However, there is still some evidence supporting the role of glial cells in pain modulation at the supraspinal level [93,101,102,103]. For example, it has been reported that microglial chemokine CXCL12 in ACC mediated the behavioral sensitization of diabetic pain. Pharmacological inhibition of microglia CXCR4 in the ACC can reduce the hyperactivity of ACC glutamatergic neurons and alleviate diabetic pain significantly in mice with diabetic pain [101]. Furthermore, a study showed that more activated microglia were found in the spinal dorsal horn and hippocampus after spared nerve injury (SNI). Pharmacological blockade or genetic ablation of microglia effectively reduced the region-specific synaptic alterations, improving neuropathic pain and memory impairments in the SNI model. Overproduction of TNF-α and microglial activation in spinal and hippocampal neurons may underlie chronic pain and memory deficits by region-dependent synaptic changes [102]. In addition, microinjection of minocycline in the RVM generated significant analgesia in rats with inflammatory pain via inhibition of descending facilitation [103].

Recently, there have been several studies suggesting that astrocytes are activated in the ACC after peripheral inflammation [104,105]. For instance, the astrocyte-mediated neuroinflammatory shifted the excitation–inhibition (E/I) balance of the ACC pyramidal neurons in the rats with chronic pain-anxiety comorbidity induced by complete Freund’s adjuvant (CFA). Proinflammatory cytokines such as TNF-α, IL-6, and IL-1β were involved in this processing [105]. Another study reported the contribution of the astrocytes to the LTP in the ACC after peripheral inflammation [104]. In any case, the relationship between the astrocytes and pain-related emotional disorders and memory impairments is also found in the hippocampus [106,107].

Although evidence of the relationship between glial cells and inflammatory pain in the CNS is increasing, the roles of glial cells in regulating functional neurons and plasticity in the pain-related cortical regions are still unclear. Therefore, considering the conflicting results of the relationship between glial cells and chronic pain in the cortical regions, more efforts are needed to further explore the basic mechanisms of chronic pain mediated by glial cells in the brain. This may help us to seek new effective targets and strategies for the treatment of chronic pain.

## 10. Possible Drug Candidates and Future Directions

As described above, accumulative studies in the field of neuropathic pain suggest several new drug targets for the future treatment of chronic pain. However, the translational development of novel medicines faces significant delays in the current economic and political environment. Concurrently, ongoing opioid misuse underscores an urgent need for precision-targeted analgesics in chronic pain management. However, the actual situation is that many pharmaceutical companies can’t overcome the great challenges and have moved away from the research and development of novel analgesics. Current pain medicines are not able to prevent or reverse brain plasticity effectively. Most medicines are based on non-selectively inhibiting excitatory synaptic transmission or enhancing inhibitory transmission. There is no doubt that such medicines will have neural side effects after the use of higher dosages or longer times to achieve pain control. For example, the popular pain target, transient receptor potential vanilloid type 1 (TRPV1), has limited effects on chronic pain, although it can be used to effectively control acute pain. To find out the question of whether TRPV1 serves as a good treatment target for chronic pain, Liu and Zhuo used the MED64 multi-channel field potential recording system to study the potential role of TRPV1 in ACC LTP [108]. Their study showed that pharmacological blockade of TRPV1 with two different types of TRPV1 antagonists (AMG9810 and SB366791) had no effect on LTP induction in the ACC of adult mice. This is in good accord with earlier reports that chronic pain was not affected in mutant mice lacking TRPV1 [109,110]. It suggests that TRPV1 may not be a potent and effective target for the treatment of chronic pain, especially at the cortical level.

As demonstrated at synaptic and circuit levels, descending positive feedback modulation possibly serves as a new potential target for designing medicines for chronic pain. Accumulative evidence indicates that descending modulation of pain mainly depends on monoaminergic pathways, including serotoninergic, norepinephrinergic, and dopaminergic descending projections [90,111,112,113,114]. Hence, the drugs that act on different 5-HT, norepinephrine or dopamine receptor subtypes may help to enhance the descending inhibition and alleviate pain responses in chronic pain patients. This is why monoaminergic drugs like serotonin/norepinephrine reuptake inhibitors (SNRIs) are useful clinically in the treatment of chronic pain [115]. Additionally, the drugs that reduce presynaptic release of glutamate, or ameliorate negative emotions, such as anxiety or depression, may be helpful to inhibit positive feedback at cortical synaptic levels [90]. It is likely that a robust descending modulatory system will be recruited to govern the development of chronic pain by the fair use of monoaminergic drugs.

**Figure 3:**
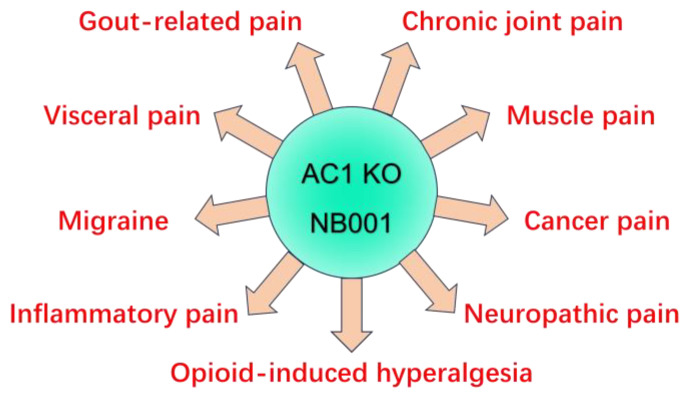
Genetic and pharmacological evidence for the contribution of calcium-stimulated AC1 in different types of chronic pain. In mice lacking AC1, behavioral responses to different sensory stimuli were significantly reduced in different chronic pain models. The selective inhibitor of AC1 NB001 shows significant analgesic effects in various models as well.

Cumulative evidence suggests that synaptic LTP that occurs at the spinal and cortical levels plays important roles in chronic pain. Drugs that selectively target synaptic LTP are more likely to treat chronic pain effectively. As mentioned above, selective inhibitors for NMDARs or mGluRs may help to reduce chronic pain, although they have some obvious cognitive side effects. AC1, a key signaling molecule for major forms of LTP in the ACC and IC, serves as a promising target for reducing chronic pain, especially those that are not driven purely by peripheral sensory inputs [116]. In addition to contributing to cortical LTPs, AC1 plays critical roles at the spinal level, and possibly in peripheral nerve terminals as well. It has been reported that AC1 is involved in 5-HT-induced excitatory synaptic facilitation in the spinal cord. The administration of a low dose of 5-HT alone, or in conjunction with the AC agonist forskolin, resulted in a sustained enhancement of excitatory synaptic transmission between primary afferent fibers and dorsal horn neurons. This process depended on the recruitment of functional postsynaptic AMPARs and AC1 activation [117]. In addition, AC1 can affect the activation of the extracellular signal-regulated kinase (Erk) and contribute to spinal sensitization, indicating a potential role of AC1 in linking upstream signals to plasticity-related gene expression and protein synthesis. Eventually, AC1 is also essential for the pairing training-induced LTP in the spinal cord [90,118]. All the data mentioned above support AC1 as a promising drug target for the treatment of chronic pain. Figure 3 updates reports of AC1 inhibitor NB001 and AC1 gene knockout in different animal models of chronic pain [116,119,120,121].

PKMζ, as another key drug target for chronic pain treatment, contributes to maintaining the expression of pain-related LTPs in the ACC, IC [53,56]. Inhibiting PKMζ activity will certainly reduce cortical LTPs, if such inhibitors like ZIP can be selectively delivered to pain synapses. Meanwhile, the potential side effects may include cognition or memory impairment due to the inhibition of LTPs also happening in the hippocampus [122,123].

Some membrane channels expressing at the CNS synapses, such as ASIC1a and HCN channel, which play crucial roles in modulating synaptic plasticity, are also good candidates for new drug targets of chronic pain [54,124,125,126]. In animal experiments, the application of ASIC1a inhibitors like tarantula or mambalgin peptide in the CNS displayed significant analgesic effects on different peripheral nociceptive stimuli [124,126]. At cortical synapses, presynaptic HCN channels are associated with hyperexcitability and the facilitation of ectopic firing in neurons. The activation of HCN channels causes presynaptic depolarization and increases the release of presynaptic neurotransmitters, which contributes to chronic pain maintenance and anxiety/depression [62,127]. A pharmaceutical study showed that the intracerebroventricular injection of HCN channel antagonist ZD7288 attenuated neuropathic pain and associated depression in SNI rats, suggesting selective inhibition of local HCN channels may be a new strategy to manage neuropathic pain and comorbidity for anxiety/depression [128].

Neuromodulators and neuropeptides (such as CGRP, oxytocin, and BDNF) have drawn plenty of attention recently due to their considerable effects in reducing different forms of chronic pain and comorbid emotional disorders. For CGRP, the antibodies as well as CGRP receptor antagonists have already been proven effective for clinical treatment of migraine and headache [129]. There are also some suggestions for the potential use of oxytocin for the management of migraine, although the mechanisms may be slightly different from that of CGRP [130,131,132]. In rodent studies, both the CGRP antagonist and oxytocin have been found to reduce cortical potentiation, which is related to chronic pain and pain-related anxiety in neuropathic pain or the migraine model [120,133]. AC1 inhibitor NB001 has also been found to alleviate migraine-related pain and anxiety in the migraine model rats [120]. One of the possible mechanisms is that AC1 contributes to CGRP-dependent LTP in the ACC and IC [134,135]. BDNF has been identified as an important neuromodulator for promoting synaptic transmission, and cellular and synaptic plasticity in the CNS and periphery [136]. Usually, it engages neuronal sensitization which contributes to the generation and maintenance of chronic neuropathic pain in the spinal cord via potentiating NMDA receptors in primary afferent terminal [137,138]. While in the ACC, BDNF was demonstrated to enhance synaptic transmission by an NMDAR-independent mechanism [139]. This process required the involvement of L-VGCC, mGluRs, AC1, CP-AMPAR, PKMζ, and tropomyosin receptor kinase B (TrkB) receptor by in vitro pharmacological experiments. Interestingly, either application of PKMζ antagonist ZIP, or AC1 antagonist NB001 blocked the BDNF-induced synaptic potentiation in the ACC, stressing the important roles of PKMζ and AC1 in chronic pain again. This finding also matched the previous study in which injections of recombinant BDNF or a viral vector synthesizing BDNF induced LTP in the ACC and sustained pain hypersensitivity [140]. In addition, exercise is found to produce long-lasting analgesic effects by normalizing BDNF upregulation and glial hyperactivity in mice with a neuropathic pain model [141]. Therefore, the application of BDNF antagonists combined with appropriate exercise may be useful to improve the treatment of chronic pain.

In conclusion, the treatment of neuropathic pain is still a serious clinical challenge. The development and maintenance of neuropathic pain depend on many elements and processes, including neurotransmitter/neuromodulator systems, neural projection networks, synaptic plastic modulation, membrane channels and related signaling pathways. The interaction among these elements and the time-dependent peripheral-central communications significantly increase the complexity of targeted therapy. Hence, here we mainly summarize the recent processes in neural projection networks, the key regulatory mechanisms of synaptic plasticity and the potential drug targets for the etiology and the therapy of chronic neuropathic pain. In addition to the ACC and IC, we think that other cortical/subcortical regions, such as the thalamus, mPFC, amygdala etc., deserve more attention and investigation as well to complete the whole projecting mapping of chronic pain in the brain. Different forms of neural plasticity and related signaling pathways in these regions are required to be further studied for a better understanding of neuropathic pain and its treatment. Selective manipulation of local neuronal LTP/LTD or synaptic tagging by application of targeted drugs or electrical/even optogenetic stimuli may be beneficial for the future treatment of neuropathic pain with few or without side effects. It is foreseeable that the targeted drugs that function only under pathological conditions but don’t affect the normal perception of acute pain would be what we need.

## Figures and Tables

**Table 1:** Key forms of cortical plasticity that are important for chronic pain.

Cortical Plasticity	Abbreviation	Key Receptors/Signals
Postsynaptic LTP	Post-LTP	NMDAR; AC1; PKMζ; GluA1;
Early phase LTP	E-LTP	NMDAR; AC1; GluA1
Late phase LTP	L-LTP	NMDAR; AC1; PKMζ
Presynaptic LTP	Pre-LTP	GluK1; AC1, HCN channel
LTD	LTD	mGluRs; AC8; GluA2
Synaptic tagging	T_LTP	AC1

LTP: long-term potentiation; LTD: long-term depression.

## Data Availability

No new data were created or analyzed in this study. Data sharing is not applicable to this article.
